# Developing a Tool for Identifying Clinical Risk From Free-Text Clinical Records: Natural Language Processing Study

**DOI:** 10.2196/64898

**Published:** 2025-09-22

**Authors:** Natasha Biscoe, Daniel Leightley, Dominic Murphy

**Affiliations:** 1 Combat Stress Centre for Applied Military Health Research Leatherhead United Kingdom; 2 King's College London King’s Centre for Military Health Research London United Kingdom

**Keywords:** natural language processing, risk, veterans, electronic patient records, self-harm

## Abstract

**Background:**

Electronic patient records are a valuable yet underused data source; they have been explored in research using natural language processing, but not yet within a third-sector organization.

**Objective:**

This study aimed to apply natural language processing to develop a risk identification tool capable of discerning high and low suicide risk among veterans, using electronic patient records from a United Kingdom–based veteran mental health charity.

**Methods:**

A total of 20,342 notes were extracted for this purpose. To develop the risk tool, 70% of the records formed the training dataset, while the remaining 30% were allocated for testing and evaluation. The classification framework was devised and trained to categorize risk as a binary outcome: 1 indicating high risk and 0 indicating low risk.

**Results:**

The efficacy of each classifier model was assessed by comparing its results with those from clinical risk assessments. A logistic regression classifier was found to perform best and was used to develop the final model. This comparison allowed for the calculation of the positive predictive value (mean 0.74, SD 0.059; 95% CI 0.70-0.77), negative predictive value (mean 0.73, SD 0.024; 95% CI 0.72-0.75), sensitivity (mean 0.75, SD 0.017; 95% CI 0.74-0.76), *F*_1_-score (mean 0.74, SD 0.033; 95% CI 0.72-0.76), and accuracy, which was measured using the Youden index (mean 0.73, SD 0.035; 95% CI 0.71-0.76).

**Conclusions:**

The risk identification tool successfully determined the correct risk category of veterans from a large sample of clinical notes. Future studies should investigate whether this tool can detect more nuanced differences in risk and be generalizable across data sources.

## Introduction

### Background

It is important to identify and manage risk in clinical populations [1]. In the United Kingdom, guidelines from the National Institute for Care Excellence require clinical mental health services to have a risk management plan in place. Individualized risk assessments must address risks to the patient—including self-harm and vulnerability to exploitation—as well as risks to others associated with the patient’s mental health condition, such as aggression and violence, and include a plan for the ongoing management of identified risks [2].

### Assessing Risk

In clinical settings, risk is typically assessed using psychometric methods and clinical interviews and patients are dichotomized into high- and low-risk groups [3,4]. However, assessing and identifying risk in clinical populations remains subjective and difficult [5]. Accurate risk assessment may be hampered by the complexity of biological, psychological, and environmental risk factors and the interplay between them. Meta-analyses have indicated that no single factor [6] or set of factors [7] is sufficiently predictive of risk to be useful in clinical risk assessments. In addition, a systematic review of 19 studies indicated that while current methods for assessing risk in veterans are sensitive to identifying risk, they also yield a high rate of false positives, which undermines the clinical utility of these methods [8].

### Novel Methods to Identify Risk

Electronic patient records (EPRs), including free-text clinical notes that document patient contact and sessions, are an underused yet rich source of data. Secondary research using natural language processing (NLP) has increasingly been used to make use of data contained in EPRs, informing clinical service planning and improving safety management [9]. NLP encompasses a range of techniques used to train algorithms to make meaning from natural speech or text, such as in speech recognition technology and can be used with large samples. Military research has also made use of these techniques to identify veterans from a clinical research database [10], as well as to categorize patients with probable posttraumatic stress disorder [11]. NLP has previously been used to identify suicide risk from EPRs of a large United Kingdom mental health service [12].

### Risks in Veterans

Veteran services may be one clinical population that could benefit from such novel risk management methods. While suicide rates are no higher than the United Kingdom general population in serving military personnel, some veteran groups, such as those below the age of 25 years, face an increased risk relative to the general public [13]. Veterans experiencing mental health difficulties may delay seeking help compared to nonveteran clinical groups [14], often waiting until they are in crisis before doing so [15]. Once in treatment, veterans may be more likely to drop out and respond less effectively to treatment [16]. In addition, high levels of comorbidity and complexity in mental health presentations are observed in treatment-seeking veterans [17], particularly those who have reached a crisis point [18]. These considerations highlight the importance of monitoring risk in veteran services and evaluating the efficacy of methods for doing so.

### This Study

This study aimed to develop an NLP framework to identify high and low risk of suicide from clinical notes of a United Kingdom veteran mental health charity. A machine learning classification framework is used to test and evaluate a range of classifiers to develop a risk identification tool.

## Methods

### Ethical Considerations

Approval for this project was granted by Combat Stress Research Committee (ref.pn2020). The study was conducted in alignment with the 2024 Declaration of Helsinki for medical research with human participants.

### Participants

The deidentified data used in this study were extracted from an EPR system managed by a third-sector charitable organization that provides mental health services to United Kingdom veterans. The charity EPR system has supported a range of research projects [19-21]. Many of these studies have aimed to answer specific clinical or population-based research questions such as the demographic profile of referrals to the charity and to develop a mobile phone app to reduce alcohol consumption.

The data used in this study were extracted from the patient EPRs, which were created by clinical staff each time they interacted with a patient or updated the patient’s file. These records provide an overview of patient interactions, treatment, risk profiles, and patient-reported concerns. Clinical risk is determined by individual clinicians, who assess and document it based on the charity’s guidelines and their professional judgment. Risk is recorded as either low or high according to these criteria.

### Procedure

There are approximately 650,000 records from 33,000 patients in the charity’s EPR. Owing to the large number of records and time constraints, we extracted a subset for the development of our risk tool. This subset (hereafter termed *risk database*) was extracted from the EPR system for patients who had a modification in their record between July 2020 and July 2022, had a clinical risk indicator and free-text summary (see quote 1 as an example), and had provided consent for their anonymized data to be used for research purposes. The extraction time frame was selected because patients must have their consent reviewed after 2 years, and due to an EPR outage that commenced in late July 2022:

Full meeting minutes can be observed in the meetings tab. Patient appears happy and consent with life, but has indicated low mood, and bouts of manic depression with some suicidal thoughts. Recommend further follow-up in two weeks.

Quote 1: synthetically generated free-text statement as an example of the type of note provided in the charity’s EPR

### How Clinical Risk Category Is Computed in the Service the Sample Is Drawn From

Every veteran referred to the charity is offered a screening call during which the frequency of suicidal ideation is assessed using item 9 of the Patient Health Questionnaire [22]. Risk status is determined by this question, along with further questions regarding intent, previous suicide attempts, current plans, and the presence of high levels of shame, guilt, impulsivity, or risk-taking behaviors. Referrals for veterans who are assessed as high risk in this regard are not accepted for a full assessment and are instead offered information about more appropriate services. During treatment, risk status is determined by subjective clinician judgment and considering the following factors: historical attempts, vague or contemplative plans, evidence of elevated alcohol or drug use, previous involvement with acute mental health services, isolation and withdrawal, evidence of frequent anger or physical aggression, impulsivity, reduction in meaningful activities, and protective factors (employment status, support network, motivation to engage in treatment, future orientation, evidence of external locus of control and self-efficacy, hope in treatment success, and triggers to mental health deterioration). A high-risk rating is assigned when the Patient Health Questionnaire-9 items 3 “feeling down depressed or hopeless” and 9 “thoughts that you would be better off dead or of hurting yourself in some way” are rated as “more than half the days” in the previous 2 weeks, as well as presence of the aforementioned risk factors.

A total of 20,342 records were retained for analysis, which are hereafter referred to as the gold standard dataset. These records included assigned clinical risk scores for 582 patients (low risk: 10,984; high risk: 9358). The clinical risk score serves as the benchmark for assessing the performance of machine learning models. Each patient may have multiple records, reflecting different points in their care. The decision to include only 582 patients was driven by resource constraints, such as limited staff time for annotating each record and the need to maintain patient privacy by processing the fewest possible number of records. Due to these considerations, a sample size calculation was not conducted.

For the development and evaluation of the risk tool, we allocated 70% of the dataset (14,239 out of 20,342 records) for training, while the remaining 30% (6103 records out of 20,342) were used for testing and evaluation. To ensure the integrity of the evaluation, patients were assigned to either the training or testing group, with no patient’s records appearing in both groups. It is important to note that there is no universally recognized method for determining the size of the training and testing groups. Therefore, our approach was guided by methodologies reported in similar studies [10,23,24].

### Generating an Annotated Word Features Dataset

In this phase, 5000 records from the training dataset, evenly divided between low risk (2500) and high risk (2500) and representing 105 patients, were annotated. To ensure balanced representation of both classes and to mitigate class imbalance during model training, we explicitly selected an equal number of records from each risk category. DL and NB developed a set of classification rules for annotating each record using an annotation protocol. This method involved categorizing words and phrases into three groups: (1) those indicating any kind of clinical risk, such as self-harm, suicidal thoughts, or suicidal intent; (2) descriptions of suicidal behavior or thoughts pertaining to individuals other than the patient; and (3) expressions that might cause confusion, such as “he was angry” or “the TV show had death.” As the researcher team analyzed each record, terms relevant terms were highlighted by the annotation software based on the aforementioned groupings. These groupings were used to extract the different feature sets used for modeling. If differences occurred, these were discussed until an agreement was reached regarding inclusion or exclusion. This systematic categorization yielded a set of word features that were used in developing and training the risk identification tool. Details of the annotation protocol and classification rules are available upon request from the corresponding author.

### Developing the Risk Identification Tool

In this study, we developed a machine learning classification framework to test and evaluate a range of machine learning classifiers.

The training dataset (defined earlier) was preprocessed to remove (1) punctuations (using regular expressions algorithm), (2) words or phrases related to another individual’s clinical risk (these were required to exactly match those in the annotated feature dataset), (3) stop words and frequently occurring terms (except words related to harm and risk), and (4) word or phrases that may cause confusion with correctly identifying self-harm and risk. The remaining features were then converted into term frequency–inverse document frequency (TF-IDF) features. The risk terms and phrases identified in the annotation phase were used for the TF-IDF features. Mutual information scores were calculated for each annotation using the mutual_info_classif function to quantify the dependency between terms and risk class. The top 500 features with the highest scores were retained for model training and incorporated into the TF-IDF features. This approach was undertaken to ensure that key phrases and terms associated with risk were given a higher prominence, whereas terms related to confusion were penalized. This approach follows a similar framework reported elsewhere [10].

The classification framework was developed and trained to identify risk with the outcome being a binary value (1=high risk and 0=low risk). A training set of 9966 records was used within the framework to select the best performing machine learning classifier to use the on the test set defined earlier. As mentioned earlier, there is no standardized approach in the literature for determining the training and testing size cutoff. This is also the case when selecting the most suitable machine learning algorithms for specific analyses, not only in the field of NLP but also in areas such as health care, agriculture, and security [12,25,26].

Therefore, in this study, a comparison was made based on 10-fold cross-validation accuracy using TF-IDF features as an input of the following machine learning classifiers (which are part of the *scikit-learn* package): support vector machines, random forest, artificial neural network, k-nearest neighbor, logistic regression, and XGBoost. It is important to note that there is sparse literature on which machine learning algorithms are best suited for specific tasks such as clinical risk detection using NLP. Therefore, algorithms have been selected based on prior use in this domain. During this initial phase, each machine learning classifier used default parameters as specified by *scikit-learn* package. The 10-fold cross-validation randomly samples the 9966 records at each “fold” to identify which parameters are suitable for the best performance. Logistic regression yielded the best performance during the 10-fold cross-validation (see Table 1 for full results).

**Table 1:** Machine learning classifier 10-fold cross-validation performance based on the training dataset of 9966 records.

Classifier	Accuracy, mean (SD)	Negative predictive value, mean (SD)	Precision (PPV^a^), mean (SD)	Sensitivity, mean (SD)	*F*_1_-score, mean (SD)
Support vector machines	0.922 (0.026)	0.898 (0.020)	0.948 (0.043)	0.900 (0.018)	0.923 (0.024)
Random forest	0.889 (0.025)	0.874 (0.043)	0.906 (0.015)	0.867 (0.043)	0.886 (0.026)
Artificial neural network	0.851 (0.014)	0.826 (0.035)	0.877 (0.017)	0.822 (0.028)	0.848 (0.012)
k-nearest neighbor	0.798 (0.031)	0.817 (0.044)	0.782 (0.031)	0.815 (0.042)	0.797 (0.028)
Logistic regression	0.977 (0.014)	0.972 (0.016)	0.983 (0.014)	0.972 (0.016)	0.977 (0.014)

^a^PPV: positive predictive value.

In addition to comparing multiple classifiers, we also considered dimensionality reduction and feature selection techniques, including SelectKBest and truncated singular value decomposition, to evaluate whether reducing the dimensionality of the feature space improved model performance. However, while these methods modestly reduced computational complexity, they did not significantly enhance classification accuracy or *F*_1_-score when compared with other approaches. Furthermore, the records were relatively short in length, resulting in low computational complexity.

### Data Analysis

All analyses were performed using Python version 3.5 with standard mathematical packages and *scikit-learn* (version 0.20.3) [26]. Machine learning classifier 10-fold cross-validation was selected as the highest accuracy with consideration of the negative predictive value, precision, sensitivity, and *F*_1_-score with each reported with SD and 95% CI with SD and 95% CI reported to represent the n-fold result.

Record characteristics were reported as the average frequency in which words, sentences, whitespaces, stop words, and nonalphanumeric across records were stratified by risk status. For evaluating the risk tool, each classifier model was tested by measuring its results against the results from the clinical risk assessment status (the gold standard testing dataset), allowing for computation of positive predictive value, negative predictive value, sensitivity, *F*_1_-score, and accuracy at a record level. In this study, positive predictive value was defined as the proportion of correctly identified true high-risk cases over the total number of true high-risk cases identified by the classifier. Negative predictive value was defined as the proportion of correctly identified true low-risk cases over the total number of low-risk cases identified by the classifier. Sensitivity was defined as the proportion of true high-risk cases identified by the classifier over the total number of actual high-risk cases (identified by each record). *F*_1_-score considers both positive predictive value and sensitivity and produces a harmonic mean, where the best value lies at 1 and the worst value lies at 0. Classifier performance was additionally assessed using the Youden index, which summarizes the trade-off between sensitivity and specificity. The Youden index is calculated as (sensitivity+specificity−1), with values ranging from 0, indicating no discriminatory power, to 1, representing perfect discrimination. Thus, it provides a useful metric for identifying the classifier threshold that maximizes both sensitivity and specificity.

## Results

### Record Characteristics

Descriptive characteristics of the gold standard dataset are reported in Table 2. These analyses indicate that high-risk records had more words (mean 472.98, SD 407.75), sentences (mean 158.88, SD: 222.47), white spaces (mean 62.80, SD 87.66) and stop words (mean 25.39, SD 50.12).

**Table 2:** Record characteristics including frequency and mean (SD) stratified by low risk and high risk.

Characteristics	Low risk (n=10,984), mean (SD)	High risk (n=9358), mean (SD)
Words	376.28 (309.17)	472.98 (407.75)
Sentences	147.17 (234.01)	158.88 (222.47)
White spaces	56.02 (85.67)	62.80 (87.66)
Stop words	25.57 (52.45)	25.39 (50.12)

### Performance

The performance of the risk tool using logistic regression is reported in Table 3 and evaluated using the testing dataset producing a positive predictive value, negative predictive value, sensitivity, *F*_1_-score, and accuracy statistics. The testing data contained 6103 records, which represented 3102 low-risk and 3001 high-risk records. Overall, the clinical risk tool had an accuracy of 0.734 (0.035; 0.712-0.756), a negative predictive value of 0.734 (0.024; 0.719-0.749), a precision of 0.737 (0.059; 0.7-0.774), a sensitivity of 0.748 (0.017; 0.738-0.758) and an *F*_1_-score of 0.741 (0.033; 0.721-0.761).

**Table 3:** Performance results for the clinical risk identification tool using logistic regression.

Performance metric	Results, mean (SD; 95% CI)
Youden index	0.734 (0.035; 0.71-0.76)
Negative predictive value	0.734 (0.024; 0.72-0.75)
Precision	0.737 (0.059; 0.70-0.77)
Sensitivity	0.748 (0.017; 0.74-0.76)
*F*_1_-score	0.741 (0.033; 0.71-0.76)

## Discussion

### Principal Findings

This study applied NLP to EPRs from a national veteran mental health charity to develop a risk identification tool and the findings demonstrate the feasibility of using a large electronic records database to classify patients into general risk categories (high or low). This is one of the growing number of studies using free-text clinical records to identify risk [12,27]. Our risk tool performed favorably in comparison to similar studies, achieving a precision of 74% in classifying risk category. One study using an NLP algorithm to identify risk reported a precision value of 60%, while another tool was able to correctly identify 92% of cases of suicide ideation and 83% suicide attempts [12]. Another machine learning algorithm was able to identify suicide attempts with a precision of between 93% and 97%.

Key to why the performance metrics of the current tool were in the middle of this range is likely the much smaller samples and more precise risk categories used in previous studies, potentially making these tools more accurate. For example, other algorithms used gold standard datasets of thousands of records rather than hundreds. Second, “suicide ideation” and “suicide attempt” are much clearer to define and dichotomize than the high and low general risk classifications in this study. Risk ratings in the EPRs encompass multiple areas of risk of harm to self and others, including substance use, homelessness, and safeguarding concerns, among other risk areas. As such the categories “high” and “low” risk are relatively general and depend on clinician interpretation of multiple factors.

Of interest in this work, we found that high-risk records contained a greater number of words compared to low-risk records and may indicate differences in documentation practices when clinicians identify increased risk. Specifically, clinicians may provide more detailed or extensive documentation when patients present higher levels of risk, reflecting more complex clinical considerations, a broader range of concerns, or increased efforts to record comprehensive management plans. This observation warrants further investigation to better understand how documentation practices may vary according to perceived risk, which in turn may inform future NLP-driven approaches for risk identification.

The relatively lower accuracy of the current tool compared to similar studies indicates that such tools should not replace clinical risk assessment by a qualified clinician. Given the importance of evaluating risk in clinical samples, only 100% accuracy would suffice. However, this study has demonstrated that risk categories can be captured by free-text clinical notes, suggesting that there may be a place for NLP algorithms in using large databases of clinical notes to find patterns in indicators of risk that may not have been considered by individual clinicians.

### Interpretability and Clinical Reasoning

An important consideration in the development of clinical decision support tools is their interpretability. That is, the extent to which the output can be explained and justified to clinicians and service providers. This is important in a mental health setting, where risk decisions have significant consequences. In this study, interpretability was a key consideration. Unlike black-box models, logistic regression (for example) allows for the direct examination of model coefficients, facilitating the identification of the most predictive features and their relative contribution to risk classification. In addition, low-complexity models can be exploited within the resource-constrained environments typical of third-sector organizations or National Health Service, where advanced infrastructure required for complex models may not be available.

Our analysis revealed that the most predictive features associated with high-risk classification included terms such as “suicidal thoughts,” “self-harm,” “hopeless,” “impulsive,” “previous attempts,” and “risk to self.” These features align closely with the clinical criteria used within the service to define and assign high-risk ratings. Conversely, terms associated with protective factors, such as “supportive family,” “engaged in treatment,” “stable,” and “improvement” were associated with low-risk classifications and contributed negatively to the model’s decision boundary.

These findings are consistent with clinical reasoning and established suicide risk frameworks. For example, indicators such as prior suicide attempts, as well as psychological markers such as hopelessness and impulsivity, are well-established predictors of suicide risk in both the general population and in veteran groups [8,23]. Similarly, the presence of social support, treatment engagement, and expressions of psychological recovery are routinely considered protective factors by clinicians when assessing risk. The ability to identify and understand which features contribute to risk classification improves trust in the algorithm and enables a mechanism for validation against clinician judgment. However, future development should continue to emphasize transparency and clinical alignment, particularly if such tools are to be integrated into routine practice within veteran mental health services.

### Uses of a Risk Tool in Veteran Services

However, the naturalistic data contained within a large EPR database was able to be exploited by this study. An advantage of developing a tool using this approach is that the methods used could transfer more easily to create similar tools on extracted EPR data from other services. Risk tools, such as the one developed in this study, may be particularly useful in improving effectiveness and efficiency of risk evaluation, which is often highly subjective and lengthy [5]. EPRs are a rich data source because they contain longitudinal, detailed information from large patient samples. Using such data could permit a more nuanced perspective of risk, which may be particularly valuable in population subgroups such as treatment-seeking veterans, who may exhibit different risk profiles compared to other groups. For example, treatment-seeking veterans face a high burden of comorbid difficulties [28] adding to the complexity of their mental health difficulties [17] and yet veterans may delay seeking support until they have reached a point of crisis [15]. Veteran groups may be more likely to underreport when asked about risk factors by clinicians [29], which highlights a potential need to use whatever data are available when identifying risk, including that contained within clinical notes.

### Strengths and Limitations

While this study was able to use a large sample to identify risk category, several limitations should be noted. First, the sample is drawn from a service that as part of its eligibility criteria does not offer treatment to veterans who are in crisis or considered at very high risk of harm to self or others. Future research should seek to understand how to capture differences in risk categories in a more fine-grained way. Second, eligibility criteria for treatment and service provision varied throughout the extraction period, including through the COVID-19 pandemic and the beginning of an EPR outage. However, the time for extraction of clinical notes was carefully considered to minimize the variation in reporting linked to these factors. It was not possible to use advanced models such as transformer-based models in this study. However, our approach aimed to replicate prior work, particularly the development of the Military Service Identification Tool [12], which focused on simpler modeling techniques. The rationale for this decision is that many National Health Service trusts and health care systems lack the necessary computational infrastructure to support advanced transformer-based architectures. Given the requirement for our approach to run behind secure health system firewalls (“behind the wire”), we prioritized models that balance performance with feasibility in real-world clinical deployments. Finally, variability and cognitive biases in clinicians’ subjective categorizations of risk are inevitable. Any transferability of the algorithms used in the current dataset would need to be adjusted to reflect different services’ reporting systems. However, the codes used to develop the current tool are malleable and unstructured.

## Abbreviations

EPR: electronic patient record
NLP: natural language processing
TF-IDF: term frequency–inverse document frequency
